# Unusual cause of domestic blast injury to the chest: Case report

**DOI:** 10.1016/j.ijscr.2024.110793

**Published:** 2024-12-27

**Authors:** Risenga Frank Chauke, Motshedi Joseph Sekgololo

**Affiliations:** Sefako Makgatho Health Sciences University, Postnet Suite 022, Private Bag X1007, Lyttelton 0140, South Africa

**Keywords:** Blast-related chest trauma, Primary and secondary survey, Management of blast related chest trauma

## Abstract

**Introduction and importance:**

Domestic blast chest injuries are because of suicide and accidents. Explosion injuries (trauma) are common in conflicts or military settings. Terrorists attacks result in injuries among the civilian population. A case of a 52-year-old African man who had an oxygen canister/grinder explode on him whilst working with it is reported. This case confirms that the mortality rate of patients with chest blast injuries who reach the hospital is low at less than 5 %.

**Case presentation:**

A 52-year-old man suffered a blast injury from an exploded oxygen canister in July 2018. The shrapnel entered his chest, shattering his ribs and causing lung lacerations. He underwent urgent thoracotomy to remove the foreign body and chest wall reconstruction. Complications included clotted haemothorax and rhabdomyolysis, leading to acute kidney injury, and requiring dialysis. He was discharged after seven weeks.

**Clinical discussion:**

Thoracic blast injuries can occur at various subcellular levels. Management involves primary and secondary surveys, followed by patient disposition. Unstable patients require immediate definitive and/or damage control treatment, while stable patients benefit from radiological investigations (chest X-ray, E-FAST, CT-scan) and physiological studies (blood gas analysis, full blood count (FBC), urea, electrolytes and creatinine (UEC) analysis, cardiac markers, and clotting profile evaluation. Definitive management is tailored to the injury's severity and location.

Blast injuries are classified into four categories: primary, secondary, tertiary, and quaternary. Our patient had secondary and quaternary injuries. The management depends on patient stability.

**Conclusion:**

Effective management protocols are essential to improve survival rates in blast-related chest trauma.

## Highlights

• DIY gone wrong
• Domestic chest blast injuries
• Primary and secondary Survey in Blastic blunt chest injuries
• Management algorithm for chest blast injuries

## Abbreviations and acronyms

E-FAST: Extended focussed assessment sonography for trauma
FBC: Full blood count
U & E: Urea, electrolytes and creatinine
ICU: Intensive care unit
BP: Blood pressure
mmHg: Millimetres of mercury
mmol/L: Millimoles per litre
pH: Potential of hydrogen - acidity
PCO2: Partial pressure of carbon dioxide
kPa: Kilopascal
FIO2: Fraction of inspired oxygen
CXR: Chest X-Ray
ECG: Electrocardiogram
VATS: Video Assisted Thoracoscopic Surgery
CT-scan: Computed tomography scan

## 1 Introduction

Domestic explosion chest injuries are because of suicide and accidents [1]. Explosion injuries are common in conflicts or military settings. Terrorists' attacks result in injuries among the civilian population. An explosion occurs when energy is released over a sufficiently small time in a sufficiently small volume to generate a pressure wave of finite amplitude traveling away from the source [2]. Thoracic blast injury usually results in microscopic destruction and subcellular-level injury [3]. Chest wall destruction and underlying organ injuries are common. Diagnostics should never delay life-saving surgical exploration in unstable patients. Management involves principles of the primary and secondary survey, which is then followed by the disposition of the patient. Urgent, life-threatening injuries are attended to first. Once the patient is stable secondary survey can be done which includes radiographic examination such as chest X-ray (CXR), CT-scan, and extended focussed assessment sonography for trauma (*E*-FAST). Physiologic investigations comprise blood gas, cardiac enzymes, full blood count (FBC), urea, electrolyte and creatinine (U & E) and clotting profile analyses. Treatment may be conservative or surgical in the form of video-assisted thoracoscopic surgery (VATS) or open thoracotomy.

## 2 Case presentation

A case of a 52-year-old African man who had an oxygen canister/grinder explode on him whilst working with it is reported. The shrapnel hit his right anterior chest, shattering the 1st to the 7th ribs and creating a huge chest wall defect.

He was transferred to the tertiary care hospital with a chest drain in situ, shrapnel stuck in the pleural space, and with a flail chest (Fig. 1A) in July 2018. On arrival, he was stable and conscious with a blood pressure (BP) of 110/82 mmHg, pulse rate of 74 beats per minute, and respiratory rate of 20 breaths per minute.

**Fig. 1A:**
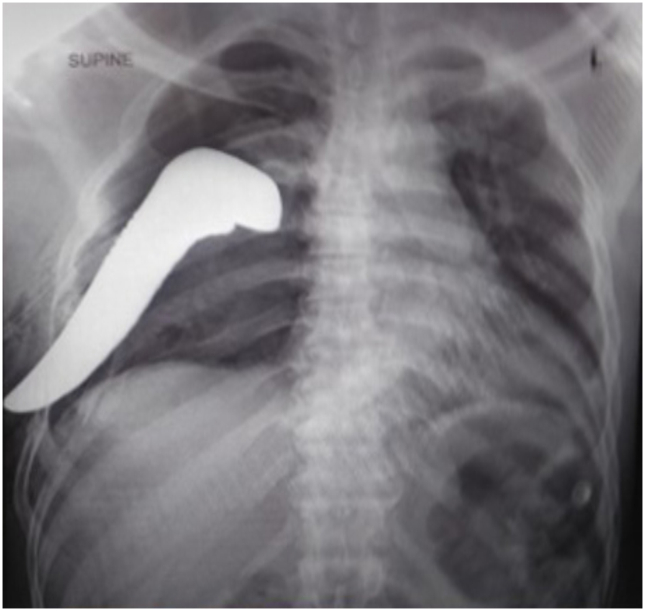
CXR with a Shrapnel stuck in pleural space pre-operatively.

The blood gas revealed a pH of 7.395 (7.350–7.450), pCO2 of 5 kPa (4.67–6.53), PO2 of 9.70 kPa (11.1–14.4), glucose of 7.5 mmol/L (3.9–5.0), and lactate was 2.1 mmol/L (0.5–1.2) at Fi0_2_ of 100 %. The patient was immediately booked to theatre and through a right posterolateral thoracotomy the pleural space was entered through the 5th intercostal space.

There was a huge defect bridging the 1st to the 4th rib. The 5th, 6th^,^ and 7th ribs had comminuted fractures and were flailing. The shrapnel (Fig. 1B) was lying against a collapsed right upper and middle lobe, with laceration of both lobes.

**Fig. 1B:**
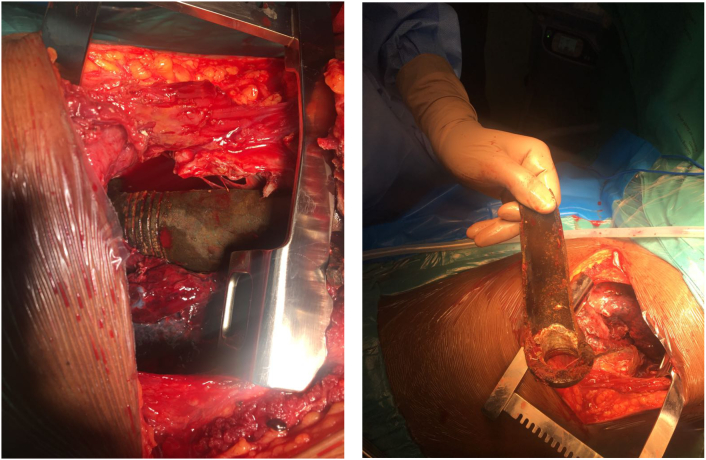
Intra-operative findings.

The pericardium and major vessels were intact after removal of the shrapnel. The bone fragments and fragments from the shrapnel were removed. The lacerations on the lung were sutured with a 4/0 Prolene busting suture. The pleural space was washed, and two drains were placed: one apical and the other basal. The chest defect was repaired with a Covidien Symbotex Marlex mesh. The lung expanded well, and the thoracotomy wound was closed in layers. The patient was then transferred to the intensive care unit (ICU) in a stable state after a bronchoscopy was done and found to be normal (Fig. 2a).

**Fig. 2a:**
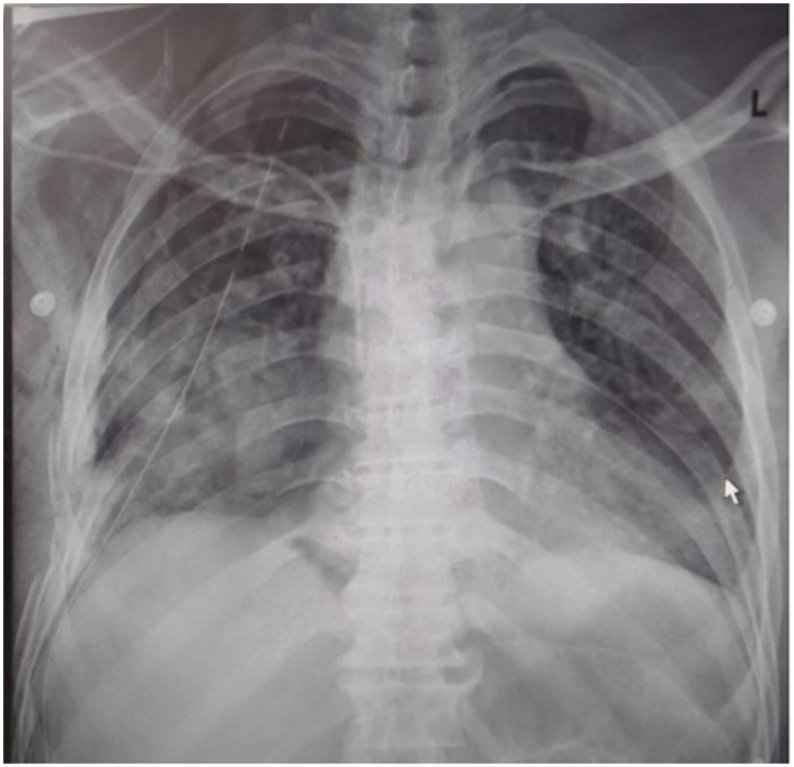
Post-op 1.

However, seven days postoperatively, the patient continued to flail and had a clotted haemothorax. A decision was then taken to take the patient back to the theatre for clot evacuation, chest wall fixation with rib plate, and removal of the Marlex mesh. A selective surgical rib Fixblu plate and screws were used to reconstruct the chest wall. Four ribs were plated and again the patient was transferred to the (ICU) in a stable state (Fig. 2b).

**Fig. 2b:**
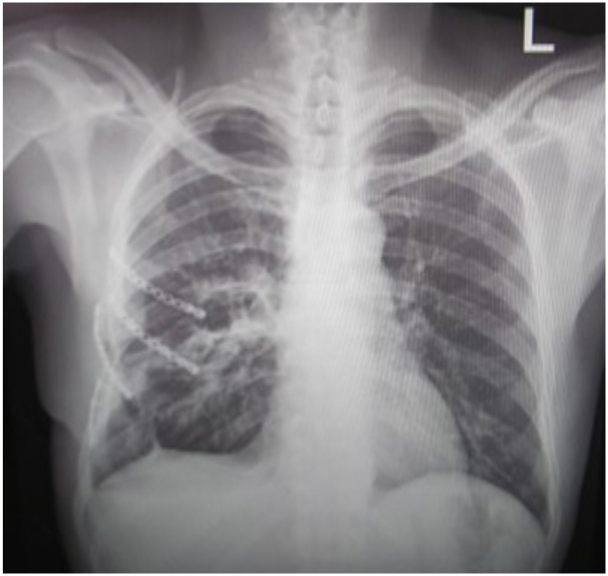
Post-op 2.

Five days later, the patient developed pre-renal failure based on kidney function assessment. Rhabdomyolysis was suspected and confirmed with myoglobin of 867μg/L (5–70) and creatine kinase of 583 U/L (22–198). He eventually developed acute kidney failure and was placed on intermittent haemodialysis.

He was eventually ventilated longer than was expected, necessitating a tracheostomy. He also contracted *Acinetobacter baumannii*, *Proteus mirabilis*, and coagulase-negative staphylococcus infections which were managed successfully, and sepsis eventually resolved with culture results that came back negative after a month.

He was in ICU for 52 days and continued with dialysis in the general ward until the acute kidney failure resolved. He was eventually discharged home after approximately two and a half months in hospital. He was followed up at the Cardiothoracic clinic. The last day of follow-up was the 15th of June 2021.

## 3 Discussion

Chest trauma is the second most common form of unintentional traumatic injury and the third cause of mortality in patients with polytrauma following abdominal and head injuries respectively. However, chest trauma mortality approaches up to 60 % in some studies therefore resulting in the highest mortality [4].

Paradoxically, the mortality rate of blast lung injury among patients who reach the hospital is low, at less than 5 % [5]. Therefore, concomitant injuries such as central nervous system injuries and polytrauma will determine the mortality.

An explosion is a violent, sudden shattering or bursting caused by the sudden fluid expansion resulting from a huge and rapid rise of pressure in air or water [1]. An explosive is any substance that can undergo exothermic oxidation turning from a solid or liquid into a gas very quickly using its energy [2]. These types of injuries are common in conflict or military settings. The mechanism of action is usually that of a blunt solid object with a limited contact surface but has a high kinetic energy and significant penetrating capacity [6]. The injury on our patient resembled that of a blunt object with high kinetic energy that penetrated the right chest causing significant damage. Shrapnel in Fig. 2b is exteriorised, revealing extensive damage/ injury to the chest wall, as well as the right upper and middle lobes. The major mechanism of wounding and mortality in the explosion is barotrauma. Barotrauma damage occurs as a result of shock waves encountering the gases of the lungs and viscera resulting in fatal blast injuries. The amount and composition of the explosive material, environment, delivery methods, or the distances between the victim and the explosive device are some of the factors that define the pattern as well as the extent of injuries caused by an explosion [1].

A stepwise description of the blast injury to the thorax and their immediate consequences follows five categories of any blast injury [1,6,7].

Primary injury occurs when the supersonic blast wave directly interacts with gas containing or hollow organs in the body, resulting in rupture. The secondary injury involves direct trauma to the victim's body caused by fragments or materials which have been energised by the explosion. The shrapnel shattered the chest wall and the underlying pulmonary parenchyma. Tertiary injury occurs when the blast wind propels the body or structure/objects, causing mass movement and impact like in cases of traumatic amputation. Quaternary injury is miscellaneous like burns, crush injury, traumatic asphyxia, and psychological disorder. Our patient developed rhabdomyolysis and eventually acute kidney failure which required dialysis. Therefore, our patient falls into the secondary and quaternary categories [1].

Lung biopsy was taken at thoracotomy and histopathology revealed haemorrhage. This was probably confirming the barotrauma effect of the explosion to the lung, apart from contusion that may have resulted from trauma on the lungs by the shrapnel.

Management follows the principle of the primary and secondary survey, which is then followed by the disposition of the patient. Urgent, life-threatening injuries are attended to first. Once the patient is stabilized, a second survey is done which is then followed by radiographic examinations. These include CXR, CT-Scan and E-FAST in some centres. E-FAST assists with pericardial and abdominal surveys. However, a CT-scan is more superior, especially in secondary surveys, because it overcomes the limitation of CXR and E-FAST, which are more valuable in primary surveys [8].

The physiology investigations which include blood gases analysis are essential, for example, in picking up low arterial gas oxygen saturation. This should also include an electrocardiogram (ECG), cardiac enzymes, FBC, U & E, and coagulation profile analyses.

Management is tailored to the resultant immediate or potentially life-threatening injuries, be it tension pneumothorax massive haemothorax, flail chest, cardiac tamponade, pulmonary contusion, trachea-bronchial injury, bronchopleural fistula, blunt cardiac injury, vascular injury, oesophageal injury or diaphragmatic injury.

This may range from simple decompression of the chest with a needle or intercostal drain to urgent surgical exploration thoracotomy. An urgent thoracotomy in a setting of blast chest trauma is associated with at least a two-fold higher mortality than penetrating trauma [8].

Urgent thoracotomy can be performed in theatre with minimal delay [9] and this may be via VATS or open thoracotomy. Other interventions include adequate analgesics, rib fixations, ventilation, and the use of supplementary oxygen, depending on the injury.

## 4 Conclusion

**Diagram 1:**
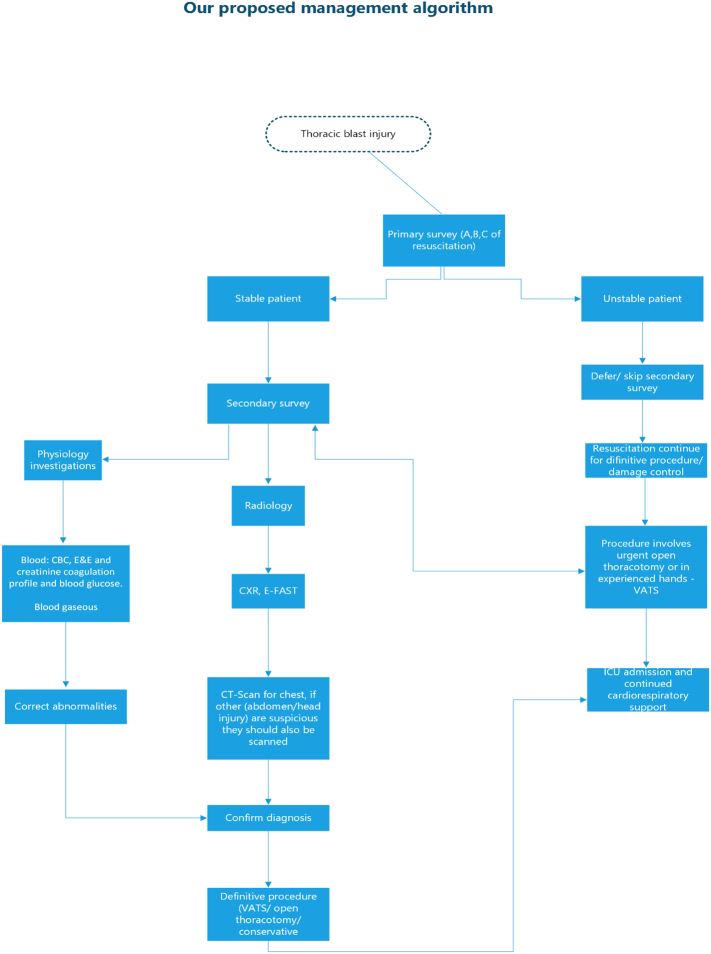
Our proposed management algorithm.

Our patient presented in a stable state, despite what is known in the literature that these types of injuries result in severe respiratory distress. The patient survived the blast injury to the chest and its resultant complications and was discharged home after having recovered from the devastating injury to the chest. Our case is a classic example of “Do it yourself” (DIY) gone wrong but survived. Management of blast chest trauma remains a challenge in clinical practice because the approach is not standardised. Damaged control surgery remains the mainstay for high-risk patients. The procedure to be undertaken to reconstruct the chest wall is the surgeon's prerogative, which may or may not be successful as was in our first procedure with mesh, and was successful in the second with plates insertion. We recommend a management algorithm for thoracic blast injury (Diagram 1).

## CRediT authorship contribution statement

Risenga Frank Chauke: Study concept and design, data collection, data analysis (Patient's hospital file), writing the paper.

Motshedi Joseph Sekgololo: Writing the paper.

## Ethical approval

Ethical approval for this study (Sefako Makgatho Health Sciences University (SMUREC/M/384/2024: J)) was provided by the Sefako Makgatho Health Sciences University Research Ethics Committee on 19 September 2024.

## Guarantor

First and corresponding Author

## Research registration number

N/A.

## Funding

None.

## Manuscript preparation

The case report was prepared in line with the SCARE guidelines [10].

## Declaration of competing interest

None.
